# Plant Vacuolar Processing Enzymes

**DOI:** 10.3389/fpls.2019.00479

**Published:** 2019-04-12

**Authors:** Barend Juan Vorster, Christopher A. Cullis, Karl J. Kunert

**Affiliations:** ^1^ Department of Plant and Soil Sciences, Forestry and Agricultural Biotechnology Institute, University of Pretoria, Pretoria, South Africa; ^2^ Department of Biology, Case Western Reserve University, Cleveland, OH, United States

**Keywords:** vacuolar processing enzyme (VPE), legumain, programmed cell death (PCD), cysteine protease, plant development

## Abstract

Plant proteomes contain hundreds of proteases divided into different families based on evolutionary and functional relationship. In particular, plant cysteine proteases of the C1 (papain-like) and C13 (legumain-like) families play key roles in many physiological processes. The legumain-like proteases, also called vacuolar processing enzymes (VPEs), perform a multifunctional role in different plant organs and during different stages of plant development and death. VPEs are similar to animal caspases, and although caspase activity was identified in plants almost 40 years ago, there still remains much research to be done to gain a complete understanding of their various roles and functions in plants. Here we not only summarize the current existing knowledge of plant VPEs, including recent developments in the field, but also highlight the future prospective areas to be investigated to obtain a more detailed understanding of the role of VPEs in plants.

## Introduction

Plant vacuolar processing enzymes (VPEs), or legumains, were named because of their role in the proteolytic processing of various vacuolar proteins (Hara-Nishimura and Nishimura, 1987). While they belong to the family of cysteine proteases, they have little sequence similarity to other cysteine proteases apart from the cysteine (Cys) and histidine (His) residues in the active site. VPEs have properties similar to animal caspases and perform limited proteolysis after asparagine (Asn) and aspartic acid (Aps) residues (Abe et al., 1993; Becker et al., 1995; Hiraiwa et al., 1999). They are also able to carry out unlimited proteolysis depending on the protein conformation of the substrate (Müntz et al., 2002). This caspase-like asparagine-specific catalytic activity was first detected in plants almost 10 years before the first successful isolation of VPEs from the cotyledons of *Vicia sativa*, then named Proteinase B (Shutov et al., 1982), and later shown to belong to the legumain family (Becker et al., 1995).

Plant VPEs are not solely expressed in seeds but also in vegetative organs (Hara-Nishimura et al., 1998; Nakaune et al., 2005). Although VPEs have been shown to play a central role in storage protein mobilization (Grudkowska and Zagdanska, 2004; Liu et al., 2018), plant development, and environmental stress responses (Christoff and Rogerio, 2014), detailed research on plant VPEs is still rather limited. To date, only a single review is available specifically focusing on their different functions in plants published 17 years ago (Müntz and Shutov, 2002) and a furthermore recent one on the contribution of a VPE to plant PCD and its role in vacuole-mediated cell death (Hatsugai et al., 2015). Recently, research relating to VPE biology has been increasing and is driven from two largely uncoupled research areas, the mammalian and the plant VPE fields (Dall and Brandstetter, 2016).

In this mini-review, we summarize the current knowledge of plant VPEs, including recent developments in the field, and highlight the future prospective areas to be investigated to obtain a better understanding of the role of VPEs in plants.

## Classification and Genomics

Evolutionary VPEs originate from prokaryote pro-legumains descending from Parabasalia and Alveolata before splitting in their separate branches of Chlorophyta and Placozoa (Shutov et al., 2012). Plant VPEs are similar to mammalian caspases, with both involved in regulating programmed cell death (PCD) pathways. In animals, one isoform is encoded and the mature enzymes are located in the cytosol. Plants, however, encode at least four functional isoforms, which are located in the vacuole (Zauner et al., 2018). Plant VPEs have been separated into three subfamilies: a seed type (β-VPE), a vegetative type (α-VPE and γ-VPE), and an uncharacterized type, only found in dicots, called δ-VPE (Müntz et al., 2002; Yamada et al., 2005). The δ-VPE group, which originated early during dicotyledonous diversification, is related to seed coat formation (Nakaune et al., 2005).

The grouping of VPEs into seed and vegetative types is related to the classification of vacuoles as either protein storing or lytic. Protein-storing vacuoles contain large amounts of defensive and storage proteins used during seed germination and growth, while lytic vacuoles contain hydrolytic enzymes (Christoff and Rogerio, 2014). Caution is necessary when using this grouping as there is no substantial sequence difference between the two types of legumains nor is the distinction enough to assign specific roles for each member of this family. This grouping also does not exclude their expression and activity in other tissues or developmental stages (Kinoshita et al., 1995; Gruis et al., 2004; Santos-Silva et al., 2012). In barley, it has been demonstrated that members of both the vegetative and seed types are almost ubiquitous and perform a multifunctional role in different organs (Julián et al., 2013).

The number of VPE genes in various plants seems to differ markedly. Four genes have been described in Arabidopsis (Kuroyanagi et al., 2005), eight in barley (Julián et al., 2013), five in rice (Hatsugai et al., 2015), and 14 in tomato (Wang et al., 2017). Recently, transcriptome sequence information has also permitted the identification of new VPE genes. The detection of additional genes by homology searches has also identified a second class of genes related to the VPEs that have a cyclization function rather than a protease function (Jackson et al., 2018). These genes have a marker of ligase activity (MLA). The MLA represents an easily identifiable marker of asparaginyl endopeptidases (AEPs) capable of post-translational cyclization of ribosomal synthesized peptides. The MLA as a diagnostic tool permits the detection of an asparaginyl endopeptidase ligase from the Violaceae plant family and has identified leads from a selection of non-cyclotide producing plant families. Although six VPE proteins were identified in the flax genome, none had the MLA region, even though flax does produce at least 21 cyclopeptides (Shim et al., 2014). As transcriptomic data have become available, the range of tissues in which a specific gene is expressed has increased, although the expression of some genes is still restricted (Ariizumi et al., 2011). In tomato, *SlVPE1* and *2* were both fruit restricted, but *SlVPE3–5* were also expressed in leaves and flowers (Ariizumi et al., 2011). As the genomic and transcriptomic data are further analyzed, the full range of activities of VPE genes with respect to plant development and response to biotic and abiotic stresses will become clearer.

## Protein Processing, Activation, and Activity

**Figure 1:**
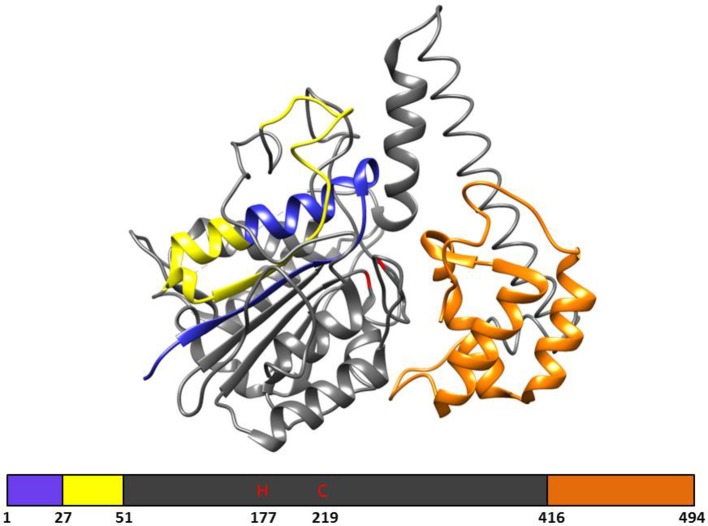
The 3D structure and primary structural organization of Arabidopsis γ-VPE. Showing the pre-prolegumain precursor with an N-terminal signal peptide (blue), the cleavable pro-peptide (yellow), and the C-terminal “pro-domain” is referred to as the Legumain Stabilization and Activity Modulation (LSAM) domain (orange). The mature protein is represented in gray and shows the catalytic histidine (H177) and cysteine (C219) amino acids. The 3D structure is based on the crystal structure (5NIJ) by Zauner et al. (2018).

In plants, VPE genes code for a pre-prolegumain precursor with an N-terminal signal peptide and a C-terminal extension (Figure 1; Müntz and Shutov, 2002) that is moved to the rough endoplasmic reticulum (RER) after translation. Removal of the signal peptide results in the inactive pro-legumains that enter the secretory pathway and are transported to the cell wall (Linnestad et al., 1998) or vacuoles (Schlereth et al., 2001). Due to the autocatalytic removal of the C- and N-terminal, the legumain pro-enzyme becomes active in the acidic pH environments of the cell wall or protein storage vacuoles (Hiraiwa et al., 1999; Müntz and Shutov, 2002). After cleavage, the C-terminal “pro-domain” will confer stability at neutral pH and modulates activity; therefore, the C-terminal “pro-domain” is referred to as the Legumain Stabilization and Activity Modulation (LSAM) domain (Zauner et al., 2018).

Substrate activity of VPEs is specific toward Ans and Asp residues, and although they have limited sequence identity, they possess similar structural and enzymatic properties to mammalian caspase-1 exhibiting YVADase/caspase-1-like cleavage activity (Hatsugai et al., 2006). Their strict cleavage specificity means that they are adapted to perform limited proteolysis of proteins; however, alteration in the substrate protein conformation caused by proteolysis with other proteases can expose additional Asn sites leading to further degradation by VPEs (Chen et al., 1998).

Recombinant γ-VPE recognizes aspartic acid as part of the YVAD sequence (VPE and caspases-1 substrates) but not others such as the DEVD sequence of caspases-3 substrates (Kuroyanagi et al., 2005). However, a VPE from *Papaver rhoeas* pollen (PrVPE1) has been described that can bind and cleave DEVD (Bosch et al., 2010) and NtTPE8 extracted from tobacco seeds, which have the ability to cleave the cathepsin H substrate FVR (Wang et al., 2018a,b) Experiments on Arabidopsis *vpe*-null mutants, which lack all four VPE gene copies, show neither VPE activity nor caspases-1-like activity (Kuroyanagi et al., 2005) and can also decrease papain-like cysteine protease activity (Cilliers et al., 2018). Certain plant VPEs have also been shown to be efficient peptide ligases and cyclases (Yang et al., 2017; Jackson et al., 2018). However, *in vitro* proof for ligase activity of *Arabidopsis thaliana* VPE isoforms is lacking (Zauner et al., 2018). VPEs have also been shown to process mitochondrial proteins in pollen (Bosch et al., 2010). VPEs also activate cysteine proteases by the removal of the I19 inhibitory domain of pre-proteases (Roberts et al., 2012). A study in *Vigna mungo* seeds confirmed that a VPE was responsible for the post-translational processing of a cysteine protease (Okamoto and Minamikawa, 1999).

Plant cysteine proteases of the papain family (C1) are inhibited by plant cystatins (Benchabane et al., 2010) and E64. Although VPEs (C13 family) belong to the cysteine protease family, they share very little sequence similarity with other cysteine proteases and are also much less sensitive to inhibition by E64 (Müntz and Shutov, 2002). However, active VPEs are inhibited by a sub-group of cystatins containing a C-terminal extension (Martinez et al., 2007; Santos-Silva et al., 2012). In soybean, only one of the 19 cystatins has this domain (van Wyk et al., 2014); similarly, only one C-terminal extended cystatin has been identified in barley (Julián et al., 2013) and rice (Christoff et al., 2016).

## Plant Development and PCD

During germination, VPEs contribute to storage protein degradation and mobilization either due to direct proteolytic degradation or through the activation of other peptidases (Schlereth et al., 2000; Zakharov and Muntz, 2004). The processing function is related to the cleavage of the C-terminal propeptides and activation of papain-like cysteine proteases as well as the N-terminal propeptides from chitinase or the processing of protease inhibitors having Asn-flanked processing sites (Kinoshita et al., 1999). VPEs have been shown to function in regulating PCD in both developmental and defense responses. PCD is a highly regulated physiological process that is essential to the development of eukaryotes. Due to the presence of the cell wall, plant cells, unlike animal cells, are not engulfed by the neighboring cells during PCD. Instead, VPEs are responsible for the collapse of the vacuole membrane resulting in the release of proteases into the cytoplasm (Hara-Nishimura et al., 2005) and initiating a proteolytic cascade leading to PCD (Hatsugai et al., 2015).

During seed development in angiosperms, the seed coat consists of two integuments of maternal tissue consisting of multiple cellular layers. During the early stages of seed development, δ-VPE is expressed in these layers resulting in limited PCD to reduce the thickness of these cell layers and form the seed coat (Nakaune et al., 2005). VPEs of barley have been shown to be involved in the degradation of maternal tissues in the seed of barley including the nucleus and pericarp (Julián et al., 2013; Tran et al., 2014), thereby also influencing seed size (Radchuk et al., 2018). VvβVPE from *Vitis vinifera* has been shown to be essential for ovule maturation and increased germination when overexpressed in Arabidopsis (Gong et al., 2018). A VPE from tobacco, *NtTPE8*, was shown to be exclusively expressed in the integumentary tapetum of tobacco seeds and downregulation of *NtTPE8* induced seed abortion (Wang et al., 2018a,b).

VPEs have also been found to mediate PCD during xylem development (Han et al., 2012) as well as during the breakdown of apical bud dominance in potato tubers (Teper-Bamnolker et al., 2012), development and senescence of root nodules (van Wyk et al., 2014; Cilliers et al., 2018), leaf and petal senescence (Kinoshita et al., 1999; van Doorn and Woltering, 2008), and pollen development (Hara-Nishimura, 2012). Hybrid lethality in tobacco has also been correlated to VPE activity and the breakdown of the vacuolar membrane (Mino et al., 2007). Two VPEs have been found to be involved in the execution of ethylene-related PCD in leaf pattern development in the lace plant (*Aponogeton madagascariensis* (Mirb.) (Rantong and Gunawardena, 2018).

## Expression Under Stress

VPEs also play a central role in the response to biotic and abiotic stresses and the likely associated change in plant hormone production. The hypersensitive response (HR) is among the known inducers of VPE expression (Zhang et al., 2010). The hypersensitive response is a form of PCD that limits pathogen development in plants and is related to plant immunity to various pathogens. PCD linked to the HR in plants following exposure to bacterial or viral pathogens could be suppressed by inhibiting VPE activity using caspase peptide inhibitors without affecting the induction of other aspects of the HR (del Pozo and Lam, 1998). Similarly, by using gene silencing, VPE deficiency prevented virus-induced HR in tobacco plants (Hatsugai et al., 2004). While the HR response is a defense against pathogen attack, toxin-induced cell death is a strategy used by pathogens during infection where compatible pathogens secrete toxins to induce host cell death and promote their growth. Kuroyanagi et al. (2005) showed that VPEs are essential for mycotoxin-induced cell death in Arabidopsis mediated by a mechanism similar to the resistance response of hypersensitive cell death.

Other known biotic inducers of VPE expression are wounding, aphid infestation, plant hormones associated with biotic stress, such salicylic and jasmonic acid, as well as nitric oxide (Julián et al., 2013; Christoff and Rogerio, 2014). In addition, plant VPEs are further considered as important components of the plant “immune” system because they can also be involved in the generation of cyclic peptides, such as Kalata B1, a peptide from the African plant *Oldenlandia affinis* and an important defense against pathogens (Craik, 2012). In rice, four of the five VPEs are responsive not only to one or several plant hormones but also to abiotic stresses (Wang et al., 2018a,b). Wounding, ethylene, and salicylic acid upregulated the expression of α-VPE and γ-VPE, while jasmonate slightly upregulated the expression of γ-VPE. In barley leaves, *HvLeg-2* (a β- or seed-type VPE) expression in both seed and vegetative tissues responds to both biotic and abiotic stimuli including salicylic and jasmonic acid, nitric oxide, and ABA in vegetative tissue, and it was also induced by gibberellic acid in seeds (Julián et al., 2013).

In general, abiotic stress, such as drought, can induce many forms of cysteine proteases, which might not be expressed during natural senescence (Khanna-Chopra et al., 1999; Martinez and Diaz, 2008; Julián et al., 2013; Cilliers et al., 2018). In our group, we also found evidence that VPEs are involved in the response to drought stress when we investigated the expression of C1 (papain-like) and C13 (VPE) cysteine proteases in soybean nodules exposed to drought conditions (Cilliers et al., 2018). A study of the cysteine protease transcriptome from soybean nodules identified a number of C1 and C13 cysteine proteases that are strongly upregulated under drought conditions. Also, by studying an Arabidopsis α-VPE deficient mutant, we found evidence that soybean α-VPE (Glyma.17G230700) might function in C1 cysteine protease maturation. VPE mutant plants had decreased C1 cysteine protease activity as well as higher biomass and protein levels under stress conditions than wild-type plants. An important role in drought tolerance was also clearly demonstrated in recent Arabidopsis work where *γ-VPE* was found to be strongly expressed in guard cells and involved in water stress response (Albertini et al., 2014). Arabidopsis plants were more drought tolerant when γ-VPE was mutated. These *γ-vpe* knock-out mutants had reduced stomatal opening, suggesting that this type of VPE has a function in the control of stomatal movements. Stomata controls photosynthesis and the water status of the plant (Nadeau, 2008). In rice, it was shown that the suppression of *OsVPE3* enhances salt tolerance by reducing vacuole rupture during PCD as well as by reducing leaf width and stomatal guard cell length (Lu et al., 2016). Stomatal closure can be triggered by pathogens, pathogen-associated molecular patterns (PAMPs), and elicitors, and VPE possibly mediates elicitor-induced stomatal closure by regulating NO accumulation in guard cells (Zhang et al., 2010), thereby playing a role in plant immunity.

## Future Research in Plant VPEs

**Figure 2:**
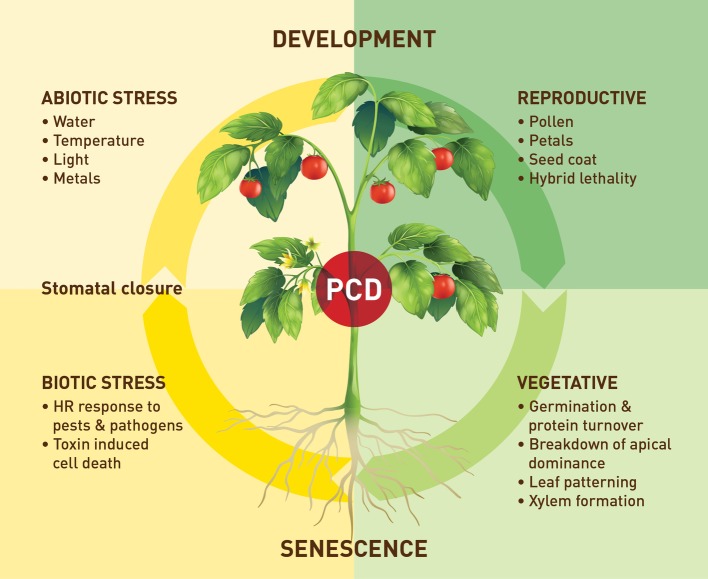
An overview of the processes in which plant VPEs are involved in.

Research on VPEs still lacks behind that on other cysteine proteases in particular the members of the C1 papain-like family. Figure 2 provides an overview of the processes in which plant VPEs are involved. Despite some recent progress in plant VPE genomics and expression, much remains to be done particularly in elucidating the role and function of VPEs, under stress conditions, and a possible role of VPEs in plant defense signaling. Therefore, more extensive VPE mutant work as well as work to silence different members of the C13 cysteine proteases (VPEs) are, in our opinion, urgently required. However, such work will also require more genomic and transcriptomic data for the identification and characterization of more VPE family members allowing to investigate the full range of activities of VPE genes with respect to plant development and response to biotic and abiotic stresses. In this regard, a better understanding of the exact role of VPEs especially in biotic and abiotic stress resistances is also needed. However, maybe any further mutant and silencing work should also include important crop species, for example, soybean and wheat (van Wyk et al., 2014; Botha et al., 2017), for which a plant transformation system is already available. Lowering VPE expression thereby limiting the maturation of papain-like cysteine proteases might provide a strategy to obtain protection against stress-induced premature senescence processes involving active papain-like cysteine proteases. It might also result in higher protein content in leaves and seeds due to lowered cysteine protease-induced protein degradation. It has been shown that co-expression of cystatins can increase recombinant protein production in tobacco (Pillay et al., 2012). Given the role that VPEs play in pathogen induced HR there may be scope to target these enzymes to improve plant expression systems for recombinant protein production. Also, the cyclization activities of some of these proteases and the identification of the associated determinants may facilitate in future work the discovery of more ligase-type AEPs and the engineering of AEPs with tailored catalytic properties. These cyclic products may have potential for both human health and pest control. A final interesting aspect to investigate in the future would be what particular role cysteine protease inhibitors play in this interaction between the two C1 (papain-like) and C13 (VPE) proteases. The type of interaction is still a largely unexplored field of study regarding time of expression and specificity of the inhibitor. For instance, the interaction between C1 proteases and their inhibitors (cystatins) is well characterized, and although VPEs have been shown to be inhibited by a cystatin with a C-terminal extension (Julián et al., 2013; Christoff et al., 2016), very little is known about the interaction of VPEs and inhibitors *in situ*.

Therefore, combining various genetic, reverse genetic, and biochemical techniques is important to identify receptors, scaffold proteins, negative regulators, and substrates of VPEs and to target genes that will help us to fully understand these plant development and defense mechanisms.

## Author Contributions

All authors contributed to the contents of the article. All authors critically reviewed the manuscript and also approved the final manuscript.

### Conflict of Interest Statement

The authors declare that the research was conducted in the absence of any commercial or financial relationships that could be construed as a potential conflict of interest.
